# The dilemma of widal test - which brand to use? a study of four different widal brands: a cross sectional comparative study

**DOI:** 10.1186/1476-0711-10-7

**Published:** 2011-02-08

**Authors:** Wafaa MK Bakr, Laila A El Attar, Medhat S Ashour, Ayman M El Toukhy

**Affiliations:** 1 Microbiology Department, High Institute of Public Health, Alexandria University, High Institute of Public Health, 165 Al Horreya avenue, Alexandria, Egypt

## Abstract

**Background:**

Serodiagnosis of typhoid fever by Widal test based on demonstrating the presence of agglutinins (antibodies) in the serum of an infected patient, against the H (flagellar) and O (somatic) antigens of *Salmonella enterica *serotype Typhi has been associated with many debates. This is why the aim of this study was to: (i) Compare the diagnostic accuracy of four different commercial kits used to perform Widal test (Remel, BioSystems, Dialab and Biotec). (ii) Compare the sensitivity and specificity of both anti-O and anti-H antibodies. (iii) Compare the validity of single versus paired serum samples with a rising titer for the diagnosis of typhoid fever.

**Methods:**

Duplicate serum samples were obtained from150 patients clinically diagnosed as typhoid fever patients. Moreover, single serum samples were obtained from 25 patients with febrile diseases other than typhoid fever. All samples were tested using the four different Widal brands and *Salmonella *Typhi IgM anti-LPS ELISA

**Results:**

**-**The results of Widal tests differed markedly using the four Widal brands in terms of sensitivity and specificity at three cut-off values of 1/80, 1/160 and 1/320. Remel brand gave the highest sensitivities and the lowest specificities and Dialab brand gave the highest specificities and the lowest sensitivities for both anti-O and anti-H antibodies at the three cut-off values.

**-**Four fold rise in the antibodies titer was not demonstrable among clinically diagnosed typhoid fever patients

-H agglutinins were less sensitive and less specific than O agglutinins

**Conclusions:**

-Widal test results showed marked discrepancies using different Widal brands. None of the serum samples of the typhoid fever patients showed four fold rise in the antibody titers. Raised O agglutinins were of slightly greater diagnostic value than raised H agglutinins.

**Significance and impact of study:**

Widal test done sequentially using two brands could be of value in typhoid fever diagnosis. Single serum sample could be used for typhoid fever diagnosis relying on anti O titer.

## Introduction

Typhoid fever, one of the enteric diseases, is endemic in Egypt [1]. Population-based studies indicated that typhoid fever incidence is 10-100/100,000 per year, with an annual peak in August [1]. An incidence of 13/100,000 persons per year was estimated in a household survey conducted in Belbis district in 2003 [2], while an incidence of 61/100,000 persons per year was estimated in Fayoum in 2002 [3].

The diagnosis of typhoid fever on clinical grounds is difficult, as the presenting symptoms are diverse and similar to those observed with other febrile illnesses [4].

Serodiagnosis of typhoid fever has been attempted since the late nineteenth century by Widal and Secard [5]. The test was based on demonstrating the presence of agglutinins (antibodies) in the serum of an infected patient, against the H (flagellar) and O (somatic) antigens of *Salmonella enterica *serotype Typhi (*S*. Typhi). While the definitive diagnosis of typhoid fever depends on the isolation of *S*. Typhi from blood, stools, urine or other body fluids [6,7], the role of the Widal test has been to increase the index of suspicion for the presence of typhoid fever by demonstrating a positive agglutination during the acute and convalescent period of infection with evidence of four fold rise in antibody titer [8-10].

Over 100 years since its introduction as a serologic means of detecting the presence of typhoid fever, the Widal test continues to be plagued with controversies involving the quality of the antigens used and interpretation of the result, particularly in endemic areas [11].

Hoffman et al. stated that the results of single Widal test, tube dilution or slide agglutination test are virtually un-interpretable unless the sensitivity and specificity of the test for the specific laboratory and patient population are known [12]. Olopenia and King stated that the value of Widal test depends upon the standardization and maintenance of the antigens to produce consistent results. They also mentioned that even since 1936 when Welch stated that no Widal test, regardless of the composition and standardization of the antigens used is infallible, and thus it is unlikely that any will be developed that will lower the validity of the isolation of the etiologic agent. Unfortunately, more than 70 years after Welch published his paper, the problems of insensitivity and non-specificity of Widal antigens continue [11].

Many authors stated that the recommended definitive interpretation of the Widal test is four fold rise in agglutinins in sera taken 7 to 10 days apart[8,13]. Four folds increase is not always demonstrable even in blood culture-confirmed cases [11,14,15].

Regarding O-agglutinin and H-agglutinin titer, Huckstep have claimed that the level of H agglutinins is unhelpful in the diagnosis of typhoid, maintaining that the H-agglutinin titer remains elevated for a longer period than the O-agglutinin titer after an episode of typhoid fever and also may rise as a nonspecific response to other infections [16]. However Brodie, Coovadia, Pang and Sommerville have proposed that the H-agglutinin titer is as useful as or more useful than the O-agglutinin titer [9,17-19]. While Parry stated that H agglutinins were less sensitive but more specific than O agglutinins and yielded better positive predictive values [20].

Hence the specific purpose of this study was to find a clue to some of these debates concerning Widal test.

### Subjects and methods

After obtaining the approval of the ethical committee of the Egyptian Ministry of Health. Patients and controls at the inpatient ward of Desouk fever hospital, kafr El-Shekh governorate, Egypt, have been invited to participate in this research. Those who accepted were recruited after obtaining their (or their parents) consents.

### Typhoid fever patients group

This group included 150 patients, 85 males and 65 females, with age ranging from 4 years to 65 years (mean age of 27.6 ± 23.33 years). They were clinically suspected of typhoid fever (suffering from continuous fever greater than 38°C in addition to headache, constipation or diarrhea).

### Hospital control group

This group included 25 patients (15 males and 10 females with mean age of 31.68 ± 19 years) with febrile diseases other than typhoid fever that have been diagnosed after both clinical examination and laboratory investigation. They included 12 patients with urinary tract infection, 5 patients with chest infection, 4 patients with brucellosis and 4 patients with measles.

### Samples collection

Paired blood samples were collected from typhoid fever patients group (300 samples). The first blood sample was obtained from 2 to 10 days from appearance of symptoms while the second sample was obtained one week interval from the first one. A single blood sample was collected from patients in the hospital control group (25 samples). Blood samples were centrifuged and sera were separated and stored at -20°C.

### Tests carried out

#### - Widal test

Widal test was performed on both first and second serum samples using the following 4 commercially available brands:

**A) Remel: **Remel stained *Salmonella *O and H suspensions, Remel Europe ltd, UK

**B) BioSystems: **Febrile serodiagnostic agglutination slides and tubes, Biosystems S A, Barcelona, Spain

**C) Dialab: **Bacterial agglutination test, Dialab, Austria

**D) Biotec: **Stained bacterial antigen suspensions, Biotec, UK

### Reagents in kits

Vials of 5 ml which contain standardized stained smooth *Salmonella *suspensions of killed bacteria which have been stained to facilitate readings of agglutination tests. These bacterial antigens were: *Salmonella *Typhi O, *Salmonella *Typhi H, *Salmonella *Paratyphi A-H and *Salmonella *Paratyphi B-H.

### Test procedure

All samples were first screened by slide agglutination technique, when proven to be positive, they were further tested by tube agglutination technique.

#### Slide agglutination technique

**- **Fifty microliters of undiluted serum were placed in a 3 cm diameter circle on a white tile

**- **One drop (50 microliters) of the appropriate well-shaken Widal suspension (both anti O and anti H) was added using the dropper provided. In performing the procedure using Remel brand, 20 microliters (instead of 50 microliters) of serum were used

- The contents were mixed by stirring for a few seconds and spread to fill the whole area of a circle on the tile. The tile was rotated slowly and agglutination was observed for one minute.

**- **Clumping within one minute was considered positive. The reaction obtained was roughly equivalent to this which would be obtained in a tube agglutination test with serum dilution of 1/80.

#### Tube agglutination technique

**Figure 1:**
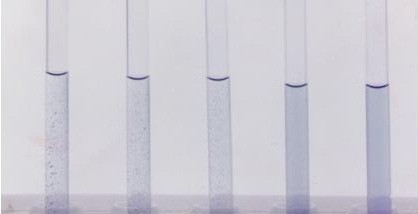
**Positive Widal test for anti-O antibodies**.

**Figure 2:**
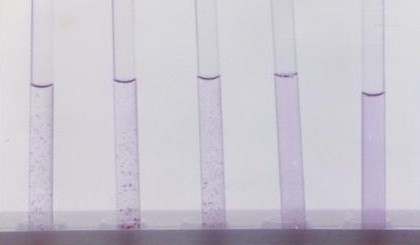
**Positive Widal test for anti-H antibodies**.

**- **The patient's sera were serially diluted (2 fold) in normal saline, starting by 1/80 to obtain dilutions of 1/80, 1/160, 1/320, 1/640, and 1/1280. One ml of each dilution was dispensed in each of 8 tubes making two rows each of 4 tubes. For the first row of tubes a drop of anti O antibodies (of each of the four used Widal brands) was added, while to the second row of tubes a drop of anti H antibodies (of each of the four used Widal brands) was added. A tube containing 1 ml saline was included as a negative control. All tubes were mixed, O suspensions were incubated at 50°C for 4 hours and H suspensions were incubated at 50°C for 2 hours. In a positive O reaction, there was a granular agglutination; while H agglutination has a characteristic floccular appearance. In a negative reaction and in the saline the appearance of the suspension should be unchanged, and show a typical swirl when the tube is flicked. The tubes were not shaken. The titer in each case is the highest tube dilution of the serum showing agglutination. Figure 1, 2

### ELISA test (*Salmonella *Typhi IgM anti-LPS ELISA BIO-QUANT INC, USA)

-In the present study detection of *S*. Typhi by culture methods was not done, instead ELISA test was performed as a comparative test to Widal on the first serum sample of 91 randomly chosen patients of the 150 clinically diagnosed typhoid fever patients.

#### Assay procedure

The test was performed according to the manufacturer instructions.

### Statistical analysis

The data were collected and statistical analysis was done. The statistical tests used namely sensitivity, specificity and accuracy. These were calculated by using the following formulas: Sensitivity is *a*/(*a *+ *c*), specificity is *d*/(*d *+ *b*), accuracy *a+d/(a+b+c + d)*, where *a *is positive ELISA and Widal test, *b *is Negative ELISA, but positive Widal test, *c *is Positive ELISA, but negative Widal test, and *d *is Negative ELISA, and negative Widal test [21].

## Results

Of the 150 clinically diagnosed typhoid fever patients included in the present study, 85 (56.7%) were males and 65 (43.3%) were females, with a mean age of 27.6 ± 23.33 years. The corresponding figures of the 25 hospital controls were 15 (60%) males and 10 (40%) females with mean age of 31.68 ± 19 years. The main presenting symptom was fever among 149 (99.3%) cases and all (100%) the 25 hospital controls. The second main presenting symptom was headache which was observed in 113 (75.3%) cases and 12 (48%) hospital controls. Constipation was more common among cases than diarrhea, it was observed in 74 (49.3%) cases, while diarrhea was observed in 62 (41.3%) cases. Regarding antibiotics intake, 140 (93.3%) of the 150 clinically diagnosed typhoid fever cases and 24 (96%) of the hospital controls received antibiotics before hospitalization.

Regarding the antibody titer assay of the first serum samples of the150 clinically diagnosed typhoid fever cases by the four used Widal brands, marked discrepancies were obtained. (Table 1)

**Table 1:** Antibody titers of the 150 clinically diagnosed typhoid fever cases in the first serum samples using 4 different Widal brands

Widal brand	Total No. of positive samples (%)		No. of samples with given reciprocal titer									
			
			80		160		320		640		1280	
	O	H	O	H	O	H	O	H	O	H	O	H
**Remel**	131 (87.34)	**141** **(94.00)**	23	**14**	42	**60**	45	**32**	19	**25**	2	**10**

**Biosystems**	129 (86.00)	**136** **(90.66)**	32	**15**	47	**60**	35	**35**	13	**18**	2	**8**

**Biotech**	125 (83.34)	**129** **(86.00)**	28	**25**	59	**55**	30	**25**	8	**20**	0	**4**

**Dialab**	103 (68.67)	**106** **(70.66)**	46	**42**	49	**41**	8	**22**	0	**1**	0	**0**

Of the 25 hospital controls diagnosed with febrile illnesses other than typhoid, only one serum sample gave positive Widal test result at a titer of 1/80 when using each of Remel, Biosystems and Biotech brands, while none of the samples reacted with Dialab brand at any titer. It should be mentioned that in Egypt the Ministry of Health and Population (MHP) consider a diagnostic titer for typhoid fever to be of 1/160 for both anti O and anti H antibodies.

Considering the difference in titers between the first and second serum samples (one week interval) of the150 clinically diagnosed typhoid fever cases none of these samples showed a three or four fold increase upon second testing for either anti-O and anti-H antibodies by any of the used Widal brands.

For comparison of the sensitivity, specificity and accuracy of the 4 used Widal brands, IgM anti-LPS ELISA test was performed as a reference test on the first serum sample of 91 randomly chosen patients of the 150 clinically diagnosed typhoid fever patients.

Table 2, 3, 4 shows the distribution of the results of 91 serum samples using the 4 Widal brands at a 1/80, 1/160 and1/320 cut-off values for anti-O antibodies in relation to their corresponding IgM anti-LPS ELISA results. Out of 91 serum samples tested by the ELISA IgM anti-LPS antibodies, 67 samples (73.62%) showed positive results. All these samples gave titers of ≥ 1/80 in the Widal test by using each of the Remel and Biosystems brands, while only 65 (97.01%) and 59 (88.05%) samples showed these titers when using the Biotech and Dialab brands respectively.

**Table 2:** Distribution of the results of 91 serum samples using the 4 Widal brands for anti-O antibodies in relation to their corresponding IgM anti-LPS ELISA results using a cut-off 1/80

Widal brand	anti -O titer ≥ 1/80	ELISA (91)				Total
			
		Positive (67)		Negative (24)		
			
		Number	%	Number	%	
**Remel**	Negative	0	0.00	13	54.16	13
	
	Positive	67	100.00	11	45.83	78

**Biosystems**	Negative	0	0.00	14	58.33	14
	
	Positive	67	100.00	10	41.66	77

**Biotech**	Negative	2	2.98	14	58.33	16
	
	Positive	65	97.01	10	41.66	75

**Dialab**	Negative	8	11.94	22	91.66	30
	Positive	59	88.05	2	8.33	61

**Table 3:** Distribution of the results of 91 serum samples using the 4 Widal brands for anti-O antibodies in relation to their corresponding IgM anti-LPS ELISA results using a cut-off 1/160

Widal brand	anti-O titer ≥ 1/160	ELISA (91)				Total
			
		Positive (67)		Negative (24)		
			
		Number	%	Number	%	
**Remel (91)**	Negative	7	10.44	20	83.33	27
	
	Positive	60	89.55	4	16.66	64

**Biosystems (91)**	Negative	11	16.41	23	95.83	34
	
	Positive	56	83.58	1	4.16	57

**Biotech (91)**	Negative	13	19.4	20	83.33	33
	
	Positive	54	80.59	4	16.66	58

**Dialab (91)**	Negative	35	52.23	23	95.83	58
	Positive	32	47.76	1	4.16	33

**Table 4:** Distribution of the results of 91 serum samples using the 4 Widal brands for anti-O antibodies in relation to their corresponding IgM anti-LPS ELISA results using a cut-off 1/320

Widal brand	anti-O titer ≥ **1/320**	ELISA (91)				Total
			
		Positive (67)		Negative (24)		
			
		Number	%	Number	%	
**Remel (91)**	Negative	27	40.29	24	100.00	51
	
	Positive	40	59.7	0	0.00	40

**Biosystems (91)**	Negative	36	53.73	24	100.00	60
	
	Positive	31	46.26	0	0.00	31

**Biotech (91)**	Negative	43	64.17	24	100.00	67
	
	Positive	24	35.82	0	0.00	24

**Dialab (91)**	Negative	65	97.01	24	100.00	89
	Positive	2	2.98	0	0.00	2

Table 5 shows the sensitivity, specificity and accuracy of the 4 Widal brands for anti-O antibodies using cut-off values of 1/80, 1/160 and 1/320 respectively using IgM anti-LPS ELISA as a reference test.

**Table 5:** Sensitivity, specificity and accuracy of the 4 Widal brand for anti-O antibodies of 91 random serum samples of the 150 clinically diagnosed typhoid fever cases using IgM anti-LPS ELISA as a reference test at three cut-off values

Using a cut-off 1/80			
**Brand**	**sensitivity**	**specificity**	**accuracy**

**Remel**	100.00%	54.16%	87.91%

**Biosystems**	100.00%	58.33%	89.01%

**Biotech**	97.00%	58.33%	86.81%

**Dialab**	88.10%	91.66%	89.01%

**Using a cut-off 1/160**			

**Remel**	89.50%	83.33%	87.91%

**Biosystems**	83.58%	95.83%	86.81%

**Biotech**	80.59%	83.33%	81.31%

**Dialab**	47.76%	95.83%	60.40%

**Using a cut-off1/320**			

**Remel**	59.70%	100.00%	70.32%

**Biosystems**	46.26%	100.00%	60.43%

**Biotech**	35.82%	100.00%	52.74%

**Dialab**	2.89%	100.00%	28.57%

Regarding the sensitivity of the 4 brands, it is clear that, each of Remel and biosystems brands showed the highest sensitivity compared to the other 2 used brands at the 3 cut-off values. However the sensitivity of these brands decreased by increasing the cut-off value it decreased from 100% at cut-off titer of 1/80, to 89.5% at cut-off titer of 1/160 and to 59.7% at cut-off titer of 1/320 for Remel brand. The corresponding figures for Biosystems brand were 100%, 83.58% and 46.26% respectively.

Regarding the specificity, Dialab brand was the most specific brand (91.66%) at the cut-off value of 1/80, however, at a cut-off value of 1/160 both Dialab and Biosystems brand were the most specific brands (95.83% for both brands). By increasing the cut-off value to 1/320, the four brands showed the same specificity (100%).

The accuracy of the 4 used brands was almost the same when using a cut-off of 1/80.

However Remel brand was the most accurate brand using the cut-off values of 1/160 and 1/320 (87.91% and 70.23% respectively)

Tables 6, 7, 8 show the distribution of the results of 91 serum sample using the 4 Widal brands at a 1/80, 1/160 and 1/320 cut-off values for anti-H antibodies in relation to their corresponding IgM anti-LPS ELISA results. It was clear from this table, that none of the used Widal brands could diagnose the 67 serum samples diagnosed by ELISA for IgM antibodies. At 1/80, 1/160 and 1/320 cut-off values the highest sensitivity (94.02%, 83.58% and 56.71%) was obtained by Remel brand and the lowest sensitivity (73.13%, 55.22% and 19.4%) was obtained by Dialab brand.

**Table 6:** Distribution of the results of 91 serum samples using the 4 Widal brands for anti-H antibodies in relation to their corresponding IgM anti-LPS ELISA results using a cut-off 1/80

Widal brand	anti-H titer ≥ 1/80	ELISA (91)				Total
			
		Positive (67)		Negative (24)		
			
		Number	%	Number	%	
**Remel**	Negative	4	5.97	2	8.33	6
	
	Positive	63	94.02	22	91.66	85

**Biosystems**	Negative	6	8.95	4	16.66	10
	
	Positive	61	91.04	20	83.33	81

**Biotech**	Negative	9	13.43	5	20.83	14
	
	Positive	58	86.56	19	79.16	77

**Dialab**	Negative	18	26.86	11	45.83	29
	
	Positive	49	73.13	13	54.16	62

**Table 7:** Distribution of the results of 91 serum samples using the 4 Widal brands for anti-H antibodies in relation to their corresponding IgM anti-LPS ELISA results using a cut-off 1/160

Widal brand	anti-H titer ≥ 1/160	ELISA (91)				Total
			
		Positive (67)		Negative (24)		
			
		Number	%	Number	%	
**Remel**	Negative	11	16.41	5	20.83	16
	
	Positive	56	83.58	19	79.16	75

**Biosystems**	Negative	13	19.4	7	29.16	20
	
	Positive	54	80.59	17	70.83	71

	
**Biotech**	Negative	18	26.86	13	54.16	31

	Positive	49	73.13	11	45.83	60

**Dialab**	Negative	30	44.77	22	91.66	52
	
	Positive	37	55.22	2	8.33	39

**Table 8:** Distribution of the results of 91 serum samples using the 4 Widal brands for anti-H antibodies in relation to their corresponding IgM anti-LPS ELISA results using a cut-off 1/320

Widal brand	anti-H titer ≥ 1/320	ELISA (91)				Total
			
		Positive (67)		Negative (24)		
			
		Number	%	Number	%	
**Remel**	Negative	29	43.28	19	79.16	48
	
	Positive	38	56.71	5	20.83	43

**Biosystems**	Negative	35	52.23	20	74.07	60
	
	Positive	32	47.76	4	25.92	31

**Biotech**	Negative	43	64.17	22	91.66	67
	
	Positive	24	35.82	2	8.33	24

**Dialab1)**	Negative	54	80.59	24	100.00	78
	
	Positive	13	19.4	0	0.00	13

Table 9 shows sensitivity, specificity and accuracy of the 4 Widal brands for anti-H antibodies using cut-off values of 1/80, 1/160 and 1/320 respectively using IgM anti-LPS ELISA as a reference test.

**Table 9:** Sensitivity, specificity and accuracy of the 4 Widal brand for anti-H antibodies of 91 random serum samples of the 150 clinically diagnosed typhoid fever cases using IgM anti-LPS ELISA as a reference test at three cut-off values

Widal Brand	sensitivity	specificity	accuracy
**Using a cut-off 1/80**			

**Remel**	94.02%	8.33%	71.42%

**Biosystems**	91.04%	16.66%	71.42%

**Biotech**	86.56%	20.83%	69.23%

**Dialab**	73.13%	45.83%	65.93%

**Using a cut-off 1/160**			

**Remel**	83.58%	20.83%	67.03%

**Biosystems**	80.59%	29.16%	67.03%

**Biotech**	73.13%	54.16%	68.13%

**Dialab**	55.22%	91.66%	64.83%

**Using a cut-off 1/320**			

**Remel**	56.71%	79.16%	62.63%

**Biosystems**	47.76%	74.07%	57.14%

**Biotech**	35.82%	91.66%	50.54%

**Dialab**	19.40%	100.00%	40.65%

Regarding the sensitivity of the 4 brands, it is clear that, Remel brand showed the highest percentages as compared to the other 3 used brands at the 3 cut-off values. However the sensitivity of this brand decreased by increasing the cut-off value, it decreased from 94.02% at 1/80, to 83.58% at 1/160 to 56.71% at 1/320.

Regarding the specificity, Dialab, brand was the most specific brand at the 3 cut-off values. The accuracy of the 4 used brands was almost the same when using a cut-off of 1/160. However, Remel and Biosystems brands were the most accurate brands using the cut-off values of 1/80 (71.42% for both).

## Discussion

Widal test has been used as an aid in the diagnosis of typhoid fever for more than a century. Since this time its value for the diagnosis of typhoid fever has been debated [11]. The specific purpose of this study was to find an answer to the following debates:

- Are different Widal brands from different manufacturers producing the same results?

-Is the Widal test sensitive and specific?

-Which type of antibodies (either O or H) is diagnostic?

-Is the test diagnostic by using a single serum sample or we should have paired sera to show a rising antibodies titer?

In the present study marked discrepancies were obtained when comparing the four used Widal brands at different cut-off values. These discrepancies in results among different brands are supported by Hoffman who stated that the results of single Widal test, tube dilution or slide agglutination test are virtually un-interpretable unless the sensitivity and specificity of the test for the specific laboratory and patient population are known [12]. This is also supported by Olopoenia and King who stated that the value of Widal test depends upon the standardization and maintenance of the antigens to produce consistent results [11].

Regarding the sensitivity and specificity of Widal test the choice of a satisfactory gold standard for diagnosis is crucial. Blood culture-positive patients as the confirmed typhoid fever cases are the usually used gold standard [19]. However, some patients with typhoid fever will be blood culture negative, particularly in areas such as Egypt, where antibiotic pretreatment is common [14,20]. Bone marrow culturing would be a better gold standard [21], but this test is not routinely performed.

In the present study both blood and bone marrow cultures were not feasible so we used IgM anti lipopolysaccharide ELISA as our reference test against which we compared the sensitivity and specificity of the used tests. Naediello et al. used IgM anti-LPS ELISA for diagnosis of typhoid where they mentioned that when both sensitivity and specificity were considered, the IgM anti-LPS test gave the best discrimination between typhoid patients and controls [22]. Also House et al. mentioned that the anti-LPS IgM ELISA was more sensitive than the Widal TO test in detecting culture confirmed typhoid cases [23].

In this study O and H agglutinins were present at a titer of 1/80 in one of the febrile hospital control groups by three of the four used Widal brands (Remel, Biosystems and Biotech) while none showed agglutinins with Dialab brand. It should be mentioned that according to the Egyptian Ministry of Health and Population (MHP) only a positive Widal titer of 1/160 for anti-O and or anti-H antibodies could be considered as a probable typhoid case, concomitant with compatible signs and symptoms of the disease [15]. So if we followed this criterion none of the 25 hospital control groups clinically diagnosed as non typhoid patients will be considered a typhoid positive case.

In this study the sensitivity and specificity of the four used Widal brands at the cut-off value of 1/160 determined by the Egyptian MHP, for both anti-O and anti-H antibodies were compared.

Regarding anti-O antibodies, Remel brand showed the highest sensitivity and accuracy of, 89.50% and 87.91% respectively, while Biosystem and Dialab brands showed the highest specificity (95.38%) each.

Regarding anti-H antibodies, Remel brand also showed the highest sensitivity of 83.58% but with decreased accuracy of 67.03%, while Dialab brand still showing the highest specificity of 91.66%.

More over, from the results of this study we were able to conclude that Remel brand gave the highest sensitivities and Dialab brand gave the highest specificities for anti-O and anti-H antibodies at the different 3 cut-off values of 1/80, 1/160 and 1/320. Indeed, the Dialab was the only brand that did not react non-specifically with the 25 sera of the control febrile cases.

Huckstep have claimed that the level of H agglutinins is unhelpful in the diagnosis of typhoid, maintaining that the H-agglutinin titer remains elevated for a longer period than the O-agglutinin titer after an episode of typhoid fever and also may rise as a nonspecific response to other infections[16]. However Brodie, Coovadia, Pang and Sommerville have proposed that the H-agglutinin titer is as useful as or more useful than the O-agglutinin titer[9,17-19]. While Parry stated that H agglutinins were less sensitive but more specific than O agglutinins and yielded better positive predictive values [20].

In this study, at the diagnostic Egyptian MHP titer of 1/160, H agglutinins were less sensitive (by all brands except Dialab) and less specific (by all brands) than O agglutinins and that a raised O agglutinins was of slightly greater diagnostic value than a raised H agglutinins.

Many authors stated that the recommended definitive interpretation of the Widal test is a four fold rise in agglutinins in sera taken 7 to 10 days apart[8,24]. None of the cases in this study showed three or four fold increase in the antibodies titer using the four Widal brands, and one or two fold increase was the common finding. This finding is supported by Hassanien et al. who stated that four fold rise, in the Widal antibody titer is uncommon and two fold rise is the common finding [25]. Satma et al. stated that four fold antibody rise is rarely demonstrated and two-to three-fold rises are more commonly seen [26]. A four fold increase is not always demonstrable even in blood culture-confirmed cases. This situation may occur due to many reasons; the early treatment of cases of typhoid fever with antibiotics, lack of antibody response in immunosuppressed patients, the delay in obtaining acute-phase sample in the natural history of the disease and finally the presence of high levels of background antibodies in a region of endemicity [11,15].

## Recommendation

- An effort must be done to establish a protocol for the standardization of the different commercially available Widal brands to ensure consistent results by different brands.

- Till the achievement of this standardization, we recommend the dual use of two Widal brands to improve the sensitivity and specificity of the test, first by screening sera with a highly sensitive brand and second by tracing false positive cases by testing positive sera in the first testing by a highly specific brand.

- If only one brand is to be used, the cut-off value of this brand must be determined to the community population.

- We recommend the use of acute phase sera using Widal test to diagnose typhoid fever cases and not rely on a 4 fold increase in the antibodies titer using paired sera.

## Competing interests

The authors declare that they have no competing interests.

## Authors' contributions

WB participated in the performance of Widal test and ELISA, interpretation of results and drafted the manuscript. LEA participated in the design of the study follow up of the work as a whole and revised the final manuscript. MA participated in the design and coordination. AET carried out the Widal test and ELISA studies and the statistical analysis. All authors read and approved the final manuscript.
